# Comparative Antiviral Activity of Remdesivir and Anti-HIV Nucleoside Analogs against Human Coronavirus 229E (HCoV-229E)

**DOI:** 10.3390/molecules25102343

**Published:** 2020-05-17

**Authors:** Keykavous Parang, Naglaa Salem El-Sayed, Assad J. Kazeminy, Rakesh K. Tiwari

**Affiliations:** 1 Center for Targeted Drug Delivery, Department of Biomedical and Pharmaceutical Sciences, Chapman University School of Pharmacy, Harry and Diane Rinker Health Science Campus, Irvine, CA 92618, USA; nibrahim@chapman.edu (N.S.E.-S.); kazeminy@chapman.edu (A.J.K.); 2 Cellulose & Paper Department, National Research Centre, 33 El-Bohouth St. former (El-Tahrir St.), Dokki, Giza P.O. Box 12622, Egypt; 3 AJK Biopharmaceutical LLC, 5270 California Ave, Irvine, CA 92697, USA

**Keywords:** antiviral, HCoV-229E, NRTIs, RNA polymerase, remdesivir, SARS-COV-2

## Abstract

Remdesivir is a nucleotide prodrug that is currently undergoing extensive clinical trials for the treatment of COVID-19. The prodrug is metabolized to its active triphosphate form and interferes with the action of RNA-dependent RNA polymerase of SARS-COV-2. Herein, we report the antiviral activity of remdesivir against human coronavirus 229E (HCoV-229E) compared to known anti-HIV agents. These agents included tenofovir (TFV), 4′-ethynyl-2-fluoro-2′-deoxyadenosine (EFdA), alovudine (FLT), lamivudine (3TC), and emtricitabine (FTC), known as nucleoside reverse-transcriptase inhibitors (NRTIs), and a number of 5′-*O*-fatty acylated anti-HIV nucleoside conjugates. The anti-HIV nucleosides interfere with HIV RNA-dependent DNA polymerase and/or act as chain terminators. Normal human fibroblast lung cells (MRC-5) were used to determine the cytotoxicity of the compounds. The study revealed that remdesivir exhibited an EC_50_ value of 0.07 µM against HCoV-229E with TC_50_ of > 2.00 µM against MRC-5 cells. Parent NRTIs were found to be inactive against (HCoV-229E) at tested concentrations. Among all the NRTIs and 5′-*O*-fatty acyl conjugates of NRTIs, 5′-*O*-tetradecanoyl ester conjugate of FTC showed modest activity with EC_50_ and TC_50_ values of 72.8 µM and 87.5 µM, respectively. These data can be used for the design of potential compounds against other coronaviruses.

## 1. Introduction

Human coronavirus 229E (HCoV-229E) is one of the seven known human coronaviruses, which include HCoV-NL63, HCoV-OC43, HCoV-HKU1, MERS-CoV, SARS-CoV-1, and SARS-CoV-2. HCoV-229E is a member of the genus Alphacoronavirus, which infects humans and bats [1]. Four of the human coronaviruses (HCoV-229E, HCoV-HKU1, HCoV-OC43, and HCoV-NL63) are associated with lower respiratory tract infections, including bronchiolitis and pneumonia [2,3] or upper respiratory tract infections characterized by rhinorrhea, nasal congestion, sore throat, sneezing, and cough that also may be associated with acute otitis media or asthma exacerbations [4]. Infection with HCoV-229E alone is most frequently associated with asymptomatic or mild disease and sometimes acute respiratory distress syndrome (ARDS) [5]. The infection is also detected with other respiratory viruses, particularly with the human respiratory syncytial virus (HRSV) [2]. These HCoV related infections may also be accompanied by asthma exacerbations or acute otitis media. Thus, exposure to HCoV-229E is low-risk for healthy adults. The HCoV-229E virus is easily accessible for the biosafety level 2 (BSL2) laboratory. Therefore, HCoV-229E may be a good initial model for the evaluation of antiviral compounds that could have potential applications against other coronaviruses, such as SARS-COV-2, the coronavirus that causes COVID-19. Although the final selected compounds are still required to be evaluated against other coronaviruses to confirm their activity.

As of May 16, 2020, according to the Johns Hopkins Coronavirus Resource Center, the human mortality of the COVID-19 pandemic infection is 313,220 people, while 4,720,197 people have been infected globally. Currently, there are no approved drugs, monoclonal antibodies, or vaccines to treat or prevent human infections caused by SARS-CoV-2. The discovery and approval of new compounds take several years. Therefore, several existing drug and potential drug candidates such as remdesivir and other antiviral agents have been considered to be repurposed as COVID-19 treatments.

**Figure 1:**
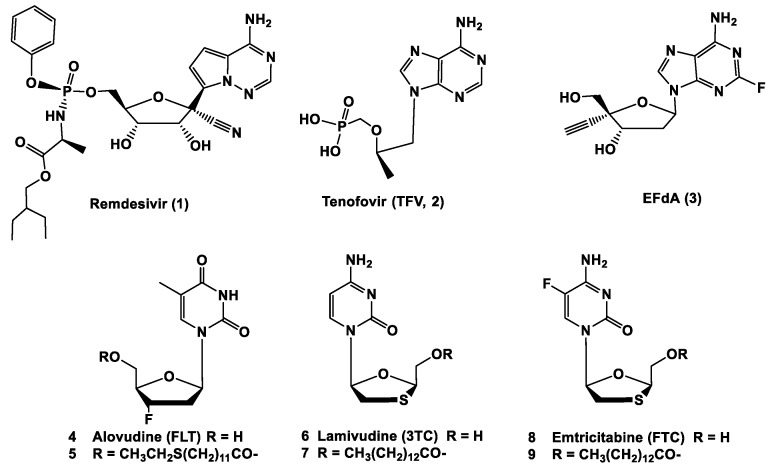
Chemical structures of remdesivir, TFV, EFdA, FLT, 3TC, FTC, and fatty acyl ester analogs of FLT, 3TC, and FTC.

Remdesivir (GS-5734, Figure 1) was developed by Gilead and found to be effective against severe acute respiratory syndrome (SARS) and Middle East respiratory syndrome (MERS) in animal models [6,7]. Remdesivir was also evaluated by Gilead for SARS-CoV-2 in early 2020. It was then used by Chinese medical researchers in patients for clinical testing in late January 2020, suggesting a favorable inhibitory effect on SARS-CoV-2 (unpublished results). Since then, several clinical trials of remdesivir have been initiated by China and the World Health Organization (WHO). Furthermore, randomized clinical trials are ongoing or planned to determine the effect on improvements in patient recovery [8]. Remdesivir is still considered one of the most promising drug candidates for the treatment of COVID-19.

On May 1, 2020, the US Food and Drug Administration (FDA stated that the potential benefits of remdesivir outweigh its known and potential risks for some patients with severe COVID-19, since a NIH study demonstrated better recovery times than with placebo [8]. The FDA issued an emergency use authorization for remdesivir for the treatment of suspected or laboratory-confirmed COVID-19 patients with severe disease.

Remdesivir is a phosphoramidite prodrug of an adenine C–nucleoside; it has a short plasma half-life (0.39 h) and is used in the IV dosage form. The compound is a broad-spectrum antiviral nucleotide prodrug that is metabolized first to the active triphosphate analog that inhibits RNA-dependent RNA polymerase (RdRp), resulting in diminished viral RNA replication [9]. There is a sequence identity among the RdRp of coronaviruses. For example, the RdRp of SARS-CoV and SARS-CoV-2 share 96% sequence identity. Therefore, drugs targeting viral RdRp proteins of SARS-CoV are likely to be effective for SARS-CoV-2 [10]. Remdesivir has been found to have ‘broad-spectrum’ anti-coronavirus activity due to its potency against previously reported coronaviruses, such as MERS-CoV, HCoV-NL63, HCoVOC43, and HCoV-229E [7,11,12]. Therefore, we assumed that compounds that are active against HCoV-229E through interfering with RdRp could also exhibit promising antiviral activity against other coronaviruses such as SARS-COV-2.

Reverse transcriptase is an enzyme in the human immunodeficiency virus (HIV) and many retroviruses that convert the RNA template to DNA. The enzyme has three enzymatic functions at the same time: (1) RNA-dependent DNA polymerase (RdRp), (2) RNase H, and (3) DNA-dependent DNA polymerase. The RNA-dependent DNA polymerase is used to synthesize a complementary DNA strand to the RNA template. After the removal of the RNA strand from the RNA–DNA hybrid double helix by RNase H, the DNA-dependent DNA polymerase completes double-stranded DNA synthesis [13]. Several nucleoside reverse transcriptase inhibitors (NRTIs) have been shown to be anti-HIV agents.

Here, we selected five NRTIs to be evaluated versue remdesivir against HCoV-229E. Tenofovir (TFV, 2) (Figure 1) is a nucleotide analog of deoxyadenosine monophosphate, with activity against HIV-1, HIV-2, and HBV [14,15]. EFdA (4′-ethynyl-2-fluoro-2′-deoxyadenosine, 3) is a highly promising potent nucleoside reverse transcriptase inhibitor (NRTI) [16,17]. Currently, EFdA is in clinical development by Merck. 3′-Fluoro-3′-deoxythymidine (FLT, alovudine, 4) and its fatty acyl ester conjugates have been used as anti-HIV agents [18,19,20]. 2′,3′-Dideoxy-3′-thiacytidine (lamivudine, 3TC, 6) [21] and 2′,3′-dideoxy-5-fluoro-3′-thiacytidine (emtricitabine, FTC, 8) [22,23] are commercially available anti-HIV agents. We have previously shown that the conjugation of certain fatty acids to the anti-HIV NRTIs, such as FLT, 3TC and FTC, enhanced activity against X4, R5, cell-associated, and/or multi-drug resistant virus when compared with their parent nucleosides [24,25,26,27].

Thus, the antiviral activity of remdesivir against HCoV-229E was compared with selected NRTIs and selected fatty acyl conjugates. The goal was to determine the antiviral activity and toxicity against HCoV-229E in an anti-coronavirus cytoprotection assay. The method could be used to select potential compounds rationally for evaluation against SARS-CoV-2.

## 2. Results and Discussion

Nine compounds were evaluated against HCoV-229E in normal human fibroblast lung cells (MRC-5) at six test concentrations. The compounds included five NRTIs (TFV (2), EFdA (3), FLT (4), 3TC (6), and FTC (8)) and three fatty acyl conjugates of FLT (5), 3TC (7), and FTC (9). We have previously reported the synthesis and evaluation of fatty acyl conjugates [19,20,24,25,26,27]. Fatty acylation of the parent nucleoside generated enhanced activity against cell-free, cell-associated, and/or multi-drug resistant virus.

**Figure 2:**
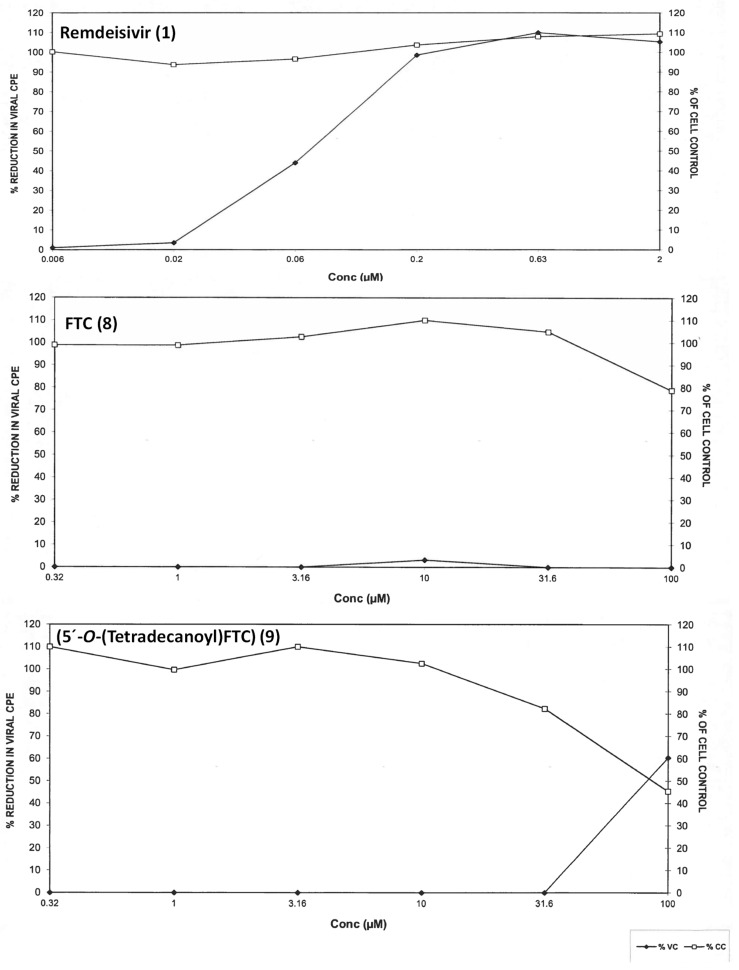
Antiviral activity and cytotoxicity of remdesivir (**1**) (EC_50_ (*n* = 3): 0.067 ± 0.012 µM, TC_50_ (*n* = 2): > 2 ± 0 µM, FTC (**8**): EC_50_ (*n* = 3) and TC_50_ (*n* = 2): > 100 µM and 5′-*O*-tetradecanoyl ester conjugate of FTC (**9**) (EC_50_ (*n* = 3): 72.8 ± 13.1 µM, TC_50_ (*n* = 2): 87.5 ± 1.34 µM against HCoV229E in MRC-5 cells. Viral cytopathic (VC) effect and MRC-5 cell cytotoxicity (CC) are shown in each graph.

The antiviral efficacy and cellular toxicity data are summarized in Table 1. The viral cytopathic effect (CPE) and cell viability were determined at each test concentration. Remdesivir exhibited EC_50_ and TC_50_ values of 0.07 µM and > 2.0, respectively, against HCoV-229E. Meanwhile, 5′-*O*-tetradecanoyl ester conjugate of FTC (**9**) demonstrated an EC_50_ value of 72.8 µM, but showed a very low calculated therapeutic index of 1.2 due to its cytotoxicity to MRC-5 cells. Compounds **3**, **5**, and **7** were cytotoxic to MRC-5 cells at TC_50,_ ranging from 45.4–55.3 µM. The parent NRTIs, **4**, **6**, and **8**, were not active against HCoV-229E at the tested concentrations. As representative examples, dose-response curves for remdesivir (**1**), FTC (**8**), 5′-*O*-(tetradecanoyl)FTC **(9)** are shown in Figure 2.

These data indicate that remdesivir acts as an antiviral agent against HCoV-229E, while anti-NRTIs agents were found to be ineffective. This could be due to the unique interaction of remdesivir with RNA-dependent RNA polymerase in coronaviruses such as HCoV-229E, while NRTIs inhibit reverse transcriptase. This enzyme has RNA-dependent DNA polymerase function. NRTIs also act as DNA synthesis chain terminators. The mode of interaction of remdesivir with RNA polymerase and the crystal structure of protein-nucleotide have not been published yet. The structure of remdesivir is unique as a nucleotide prodrug, with the presence of a nitrile group at the 1′ position and both 3′ and 4′-hydroxyl groups, leading to strong binding to RNA polymerases that differentiates this compound from the other nucleoside analogs represented here. The structure of RNA-dependent RNA polymerase of SARS-COV-2 was recently published [28]. Further structural modification of anti-HIV nucleosides could incorporate some functional groups for binding to RNA polymerases, and be used for more rationale-based antiviral drug design against coronaviruses. Furthermore, the determination of the crystal structure of remdesivir in terms of its binding with RdRp will provide insights into understanding the critical functional groups for the binding and design of the next generation of nucleoside-based inhibitors with higher binding affinities.

## 3. Conclusions

A series of anti-HIV nucleosides and their fatty acyl derivatives were compared with remdesivir for antiviral activity against HCoV-229E in MRC-5 cells. Among all the compounds, remdesivir was found to be potent, with an EC_50_ value of 0.07 µM and a therapeutic index of more than 28.6 µM. The 5′-*O*-(tetradecanoul) ester derivative of FTC showed modest activity, with an EC_50_ value of 82 µM. In general, NRTIs did not show comparable activity against HCoV-229E, compared to remdesivir. This work advances scientific knowledge in the area of the testing of antiviral compounds and the activity of anti-HIV drugs against coronaviruses. This information could also be used to design compounds that are potentially effective against other coronaviruses, such as SARS-COV-2.

## 4. Materials and Methods

### 4.1. General Reagents

The anti-HIV nucleosides were purchased from Euro Asia Trans Continental (Bombay, India). The synthesis and evaluation of fatty acyl conjugates were conducted according to the previously reported procedures in our laboratory [19,20,24,25,26,27]. The compounds were solubilized at 40 mM in 100% DMSO immediately before assay set up. The test materials were evaluated using a high test concentration of 100 µM and five serial half-logarithmic dilutions in triplicate for the antiviral assay. The compounds were diluted to 200 µM (5 µL of 40 mM stock) in a drug dilution tube containing 995 µL of assay medium. Three hundred twenty microliters (320 µL) of the 200 µM solution was transferred to 680 µL of assay medium (half-log dilution) for a total of five serial dilutions. One hundred microliters of each concentration were added in triplicate wells for efficacy, duplicate wells for cytotoxicity, and a single well for colorimetric evaluation. Remdesivir was purchased from MedChem Express (Monmouth Junction, NJ) and evaluated as a positive control compound in the antiviral assay.

### 4.2. Anti-Coronavirus Cytoprotection Assay

#### 4.2.1. Cell Preparation

The viral assay protocols were approved by the Institutional Biosafety Committee (IBC) at Imquest Biosciences. MRC-5 cells were obtained from ATCC (CCL-171) and passaged in the DMEM medium supplemented with FBS (10%), penicillin (100 U/mL), sodium pyruvate (1 mM), l-glutamine (2 mM), streptomycin (100 µg/mL), and NEAA (0.1 mM) using T-75 flasks before use in the antiviral assay. Preceding the assay, the cells were divided into 1:2 to make sure they were in an exponential growth phase at the time of infection. The quantification of total cells and viability were conducted using a hemocytometer and Trypan Blue dye exclusion.

#### 4.2.2. Virus Preparation

Coronavirus 229E (HCoV-229E) was obtained from ATCC (VR-740) and grown in MRC-5 cells (ATCC# CCL-171) for the production of a stock virus pool. A pretitered aliquot of the virus was removed from the freezer (−80 °C). The aliquot was allowed to thaw slowly to room temperature in a biological safety cabinet. The virus was resuspended and diluted into assay medium (DMEM supplemented with 2% heat-inactivated FBS, penicillin (100 U/mL), l-glutamine (2 mM), streptomycin (100 µg/mL)), in such a way that 100 µL of virus was added to each well. This amount was determined to result in 85 to 95% cell death at 6 days postinfection. Each plate contained virus control wells (cells plus virus), triplicate cell control wells (cells only), drug toxicity wells according to the compound (cells plus drug only), as well as triplicate experimental wells (drug plus cells plus virus).

#### 4.2.3. Efficacy and Toxicity

A cell viability of more than 95% for the cells was utilized in the assay. The cells were resuspended at 3 × 10^3^ cells per well in the tissue culture medium. Then, the cells (a volume of 100 µL) were added to flat-bottom microtiter plates. The microtiter plates were incubated at 37 °C/5% CO_2_ overnight to allow cell adherence to occur. After incubation of the test compounds at 37 °C in a 5% CO_2_ incubator for six days, the plates were stained with the tetrazolium dye 2,3-bis(2-methoxy-4- nitro-5-sulfophenyl)-5-[(phenylamino)carbonyl]-2H-tetrazolium hydroxide (XTT). XTT tetrazolium is known to be metabolized by the mitochondrial enzymes of metabolically active cells into a soluble formazan product. This reaction makes it possible to promptly perform quantitative analyses of the inhibition of virus-induced cell killing by antiviral test substances. XTT was prepared daily as a stock solution of 1 mg/mL in RPMl1640. Phenazine methosulfate (PMS) solution was prepared in PBS (0.15 mg/mL) and stored in the dark at −20 °C. XTT/PMS stock was prepared immediately before each use by adding a volume of 40 µl of PMS per ml of XTT solution. XTT/PMS (50 µL) was added to each well of the plate, and the plate was reincubated for 4 h at 37 °C. Plates were sealed with adhesive plate sealers. Then, they were shaken gently or inverted several times to mix the soluble formazan product. The plates were read spectrophotometrically at 450/650 nm with a Molecular Devices Vmax plate reader (Molecular Devices, LLC., San Jose, CA, USA). 

#### 4.2.4. Data Analysis

The raw data were collected from Softmax Pro (version 7, Molecular Devices, LLC, San Jose, CA, USA) and imported into a Microsoft Excel Xlfit4 (version 4, from IDBS lab, Boston, MA, United States) spreadsheet for analysis using four-parameter curve fit calculations. The antiviral activity and toxicity with graphic representation of the data were provided in a Plate Analysis Report (PAR) summarizing the individual compound activity and a summary table of calculated TC_50_, EC_50_, and Tl_50_ values. The graphical presentation shows the percentages of cell viability and of reduced viral Viral cytopathic effect (CPE) at each test concentration.

## Figures and Tables

**Table 1:** Antiviral activity and cellular toxicity against CoV229E in MRC-5 Cells.

		MRC-5/HCoV-229E	
Compound	EC_50_^a^ (µM)	TC_50_^b^ (µM)	Therapeutic Index ^c^
**Remdesivir (1)**	0.07	> 2.0	> 28.6
**TFV (2)**	> 100	> 100	-----
**EFdA (3)**	> 55.3	55.3	-----
**FLT (4)**	> 100	> 100	-----
**5′-*O*-(12-thioethydodecanoyl)FLT (5)**	> 45.4	45.4	-----
**3TC (6)**	> 100	> 100	-----
**5′-*O*-(tetradecanoyl)3TC (7)**	> 47.5	47.5	-----
**FTC (8)**	> 100	> 100	-----
**5′-*O*-(tetradecanoyl)FTC (9)**	72.8	87.5	1.20

^a^ Effective concentration that reduced 50% of viral cytopathic effect measured from triplicate data points; ^b^ Toxic concentration that killed 50% of MRC-5 cells measured in duplicate data points; ^c^ TC50/EC50.
